# Deubiquitinase OTUD7B is a potential prognostic biomarker in diffuse large B-cell lymphoma

**DOI:** 10.7150/jca.65835

**Published:** 2022-01-04

**Authors:** Shi Qiu, Yizhen Liu, Ailing Gui, Zuguang Xia, Wen Liu, Juan J. Gu, Ji Zuo, Ling Yang, Qunling Zhang

**Affiliations:** 1 Department of Cellular and Genetic Medicine, School of Basic Medical Sciences, Fudan University, Shanghai, 200032, China; 2 Department of Medical Oncology, Fudan University Shanghai Cancer Center; Department of Oncology, Shanghai Medical College, Fudan University, Shanghai 200032, China; 3 Departments of Medicine, Roswell Park Cancer Institute; Buffalo, NY, USA

**Keywords:** OTUD7B, Diffuse large B-cell lymphoma, Rituximab, Chemotherapy, Prognosis

## Abstract

OTUD7B is a deubiquitinase and has been reported as a prognostic factor in various solid tumors. However, its prognostic value in lymphoma patients remains unclear. We detected OTUD7B expression levels in 160 diffuse large B-cell lymphoma (DLBCL) tissue samples by immunohistochemistry, and analyzed correlations between its expression and clinic-pathologic parameters as well as clinical outcomes. We also investigated association between OTUD7B expression and chemotherapeutic drugs anti-tumor activity in vitro. We found that OTUD7B overexpressed in 129 (80.6%) cases, and patients with overexpression of OTUD7B experienced better overall survival comparing to those with OTUD7B low expression (*P*=0.021). Multivariate Cox regression analysis illustrated that OTUD7B was an independent prognostic indicator. In DLBCL cell lines, we found that Chidamide could up-regulate OTUD7B in several DLBCL cell lines, and also had synergistic effect with doxorubicin at low concentration. Our data illustrated that OTUD7B deficiency is a negative predictor of clinical outcome, and might be a potential therapeutic target in the treatment of diffuse large B-cell lymphoma.

## Introduction

Diffuse Large B-cell lymphoma (DLBCL) is the most common subtype of lymphoma and most patients can be cured under immune-chemotherapy 1. One third of patients will relapse and acquired resistance to chemotherapy and turned to incurable. The identification of biomarkers of clinical behavior may aid in the implementation of novel therapeutic strategies.

OTUD7B is a member of the A20 family of DUBs, it is a deubiquitinase and regulates inflammation, T-cell activation, as well as some signaling pathways 2-5. Recent researches showed its potential role in genome maintenance and cancer cell proliferation. Accumulated evidence indicated that low expression of OTUD7B associated with inferior survival in various solid tumors, such as breast cancer, hepatocellular carcinoma, and lung cancer 6-8. The role of OTUD7B expression in lymphoma patients remains unclear. Our present study aims to evaluate the prognostic significance of OTUD7B in patients with DLBCL and to characterize its molecular mechanisms in the treatment of this kind of disease.

## Materials and Methods

### Patients and samples

A total of 160 patients were included in this retrospective study who were newly diagnosed DLBCL and treated in Fudan University Shanghai Cancer Center, Shanghai, China from April 2009 to December 2018. The inclusion criteria for this study were as follows: 1) 18-75 years old; 2) histological confirmed, newly-diagnosed diffuse large B-cell lymphoma; 3) Eastern Cooperative Oncology Group (ECOG) status of 0-1; 4) hospitalized patients and received the whole cycle treatment in Fudan University Shanghai Cancer Center. Exclusion criteria included: 1) history of malignancies; 2) prior history of anti-cancer treatment before biopsy; 3) inadequate tissue specimen for research. This study was approved by the Institutional Review Board of the Fudan University Shanghai Cancer Center. And all patients signed informed consent forms for reviewing their medical records and research. All patients were pathologically confirmed as DLBCL through surgical or biopsy samples, and all pathological results were reviewed by experienced pathologists in pathology department of Fudan University Shanghai Cancer Center. Survival data were available with a median follow-up of 2,338 days (range 82~3,527 days).

### Immunohistochemistry (IHC)

Formalin-fixed, paraffin-embedded tissue slides were deparaffinized and rehydrated firstly. After the antigen retrieval which has been done by boiling slides in EDTA antigen retrieval solution (pH 8.0) for 20 min, the slides were immersed in 3% hydrogen peroxide solution for 15 min and blocked with 1% bovine serum albumin (BSA) for 30 min at room temperature. Then the slides were incubated with primary antibody against OTUD7B (Proteintech, IL, USA) overnight at 4℃ at 1:150 dilutions, and incubated with secondary antibody for 1 hour at room temperature. The slides were washed three times with PBS (pH 7.4) between each incubation step and the slides with no primary antibodies added served as negative controls. At last, the slides were visualized using the Dako Envision System (Dako, Glostrup, Denmark) following the manufacturer's instructions and subsequently counterstained with hematoxylin.

### Immunohistochemical assessment

Protein expression levels were determined on the basis of staining intensity and the percentage of immunoreactive cells. Tumors were considered positive when at least 20% of tumor cells expressed OTUD7B. The expression was evaluated in different areas with at least 1000 tumor cells were counted. Assessment was done by three pathologists without prior knowledge of the clinical features or follow-up data of the patients.

### Cell Lines and culture

The human DLBCL cell line RL, DB, RIVA and U2932 were purchased from American Type Culture Collection (ATCC, Manassas, VA). RL-4RH cell line was generated as previously described 9.

All the cell lines were maintained in RPMI 1640 supplemented with 10% heat-inactivated fetal bovine serum, 100U/ml penicillin and 100 g/ml streptomycin, maintained in 5% CO2 at 37℃.

### MTT Assay

Cells were set in 96 well plates at a cell density of 1×10^5^ cells/ml and were exposed to different drugs with concentration gradient for 72 hours. The concentration of doxorubicin was 0.005, 0.01, 0.015, 0.02, 0.025, 0.05, 0.1, 0.3, 0.5μmol/L. The concentration of Chidamide was 0.25, 0.5, 1, 2.5, 5, 7.5, 10μmol/L. The concentration of Arsenic trioxide was 0.05, 0.125, 0.25, 0.02, 0.5, 1.25, 2.5, 3.75, 5μg/mL. The concentration of all-trans-retinoicacid was 5, 10, 20, 40, 60μmol/L. The concentration of 5-Azacytidine was 0.05, 0.1, 0.25, 0.5, 1, 5, 10μg/ml. Then 20 μl of 0.5 mg/ml MTT (Thiazolyl Blue Tetrazolium Bromide, Sigma) was added to each well and incubated at 37° C for 4 h. In the end, the supernatant was disposed and 150 μl DMSO (dimethyl sulphoxide, D8418, Sigma) was added to stop the reaction. The absorbance values (OD570 nm) were measured using a spectrophotometer.

### Western blot

Cells were set in 10 cm dishes at a concentration of 3×10^5^ in 10ml, then treated with doxorubicin, Chidamide, Arsenic trioxide, All-trans-retinoicacid and 5-Azacytidine for 48h, RPMI 1640 was added as a control. Each cell lines were treated with the IC50 of each drug, in detail, doxorubicin was 0.04μmol/L for RL and RIVA, was 0.1μmol/L for DB and U2932. Chidamide was 0.8, 1, 1.4 and 3μmol/L for RIVA, DB, U2932 and RL. Arsenic trioxide was 0.9, 1, 1 and 1.2μg/mL for U2932, RIVA, DB, and RL. All-trans-retinoicacid was 30, 35, 50 and 60μmol/L for DB, U2932, RL and RIVA. 5-Azacytidine was 0.5, 1, 1 and 10μg/ml for RL, DB, U2932 and RIVA.

Cells were lysed in 200 μl RIPA lysis buffer and the supernatant were added with same volume of 2× SDS loading buffer, then boiling for 5 minutes. The protein were separated by 10% SDS-PAGE and transferred to PVDF membranes (Millipore,Temecula, CA, USA). The membrane was incubated with rabbit monoclonal antibody against OTUD7B (Proteintech, IL, USA) at 1:2000 dilutions overnight at 4 °C. After washed 3 times with TBST, the membrane was incubated with horseradish peroxidase linked secondary antibodies for 1 hour at room temperature. Proteins were visualized using Tanon full-automatic light detecting system with the BeyoECL Star (Ultra hypersensitive ECL chemiluminescence kit). Rabbit polyclonal to GAPDH was used as loading control. All the data were confirmed by three individual experiments.

### Statistical analysis

All analyses were performed using PASW Statistics 18 (SPSS Inc., Chicago). Correlation between the OTUD7B expression and clinical variables were tested by Pearson Chi Square test. Kaplan-Meier survival curves were constructed for survival analyses, and differences were tested by the log-rank test. Overall survival was defined as the time between the date of biopsy and the date of death or the date of last contact. Progression-free survival (PFS) refers to the period from the beginning of treatment to the observed progression of the disease or the occurrence of death for any reason. The data of patients alive at the end of the study were censored. All *P* values were two-sided, and the results were considered significant if P < 0.05.

## Results

### Demographic and baseline patient characteristics

Among 160 patients who were all hospitalized, median age was 51 years (range, 19 to 73 years) at the time of diagnosis and 89 (55.6%) were male. According to Ann Arbor staging criteria, 98 patients (61.3%) had stage I/II diseases, and 62 patients (38.7%) had stage III/IV diseases. 108 patients (67.5%) were IPI 0 or 1 and 52 patients (32.5%) were IPI 2 or more. ECOG status was 0-1. Concerning of cell of origin, 64 patients (45.1%) were germinal center B-cell lymphoma (GCB) and 78 (54.9%) were non-germinal center B-cell lymphoma (non-GCB) according to Han's algorithm. 21 patients (13.1%) had more than one extranodal lesions (Table 1).

### OTUD7B expression in lymphoma samples

**Figure 1:**
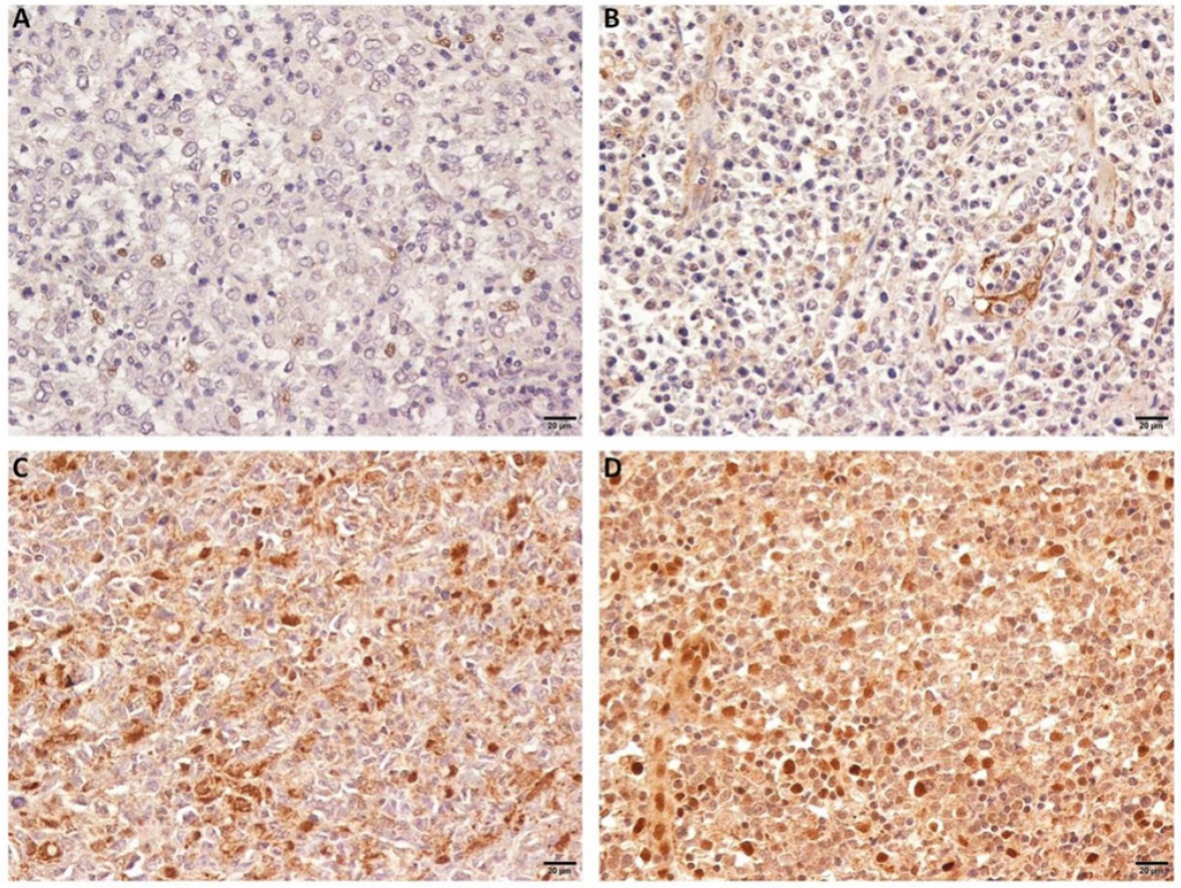
** OTUD7B expression in DLBCL patients.** Representative immunohistochemical staining of OTUD7B with negative (A,B) and positive expression (C, D).

We observed that OTUD7B was primarily localized in the nucleus and on cytoplasm. The protein strongly expressed in 129 (80.6%) cases. Figure 1 showed representing images of negative and positive expression of OTUD7B. Overexpression of OTUD7B was observed more frequently in patients with non-GCB subtype than GCB subtype (53% vs. 47%), but there was no statistically difference (*P*=0.31).

### The association of OTUD7B expression with clinico-pathological features and treatment outcome

**Figure 2:**
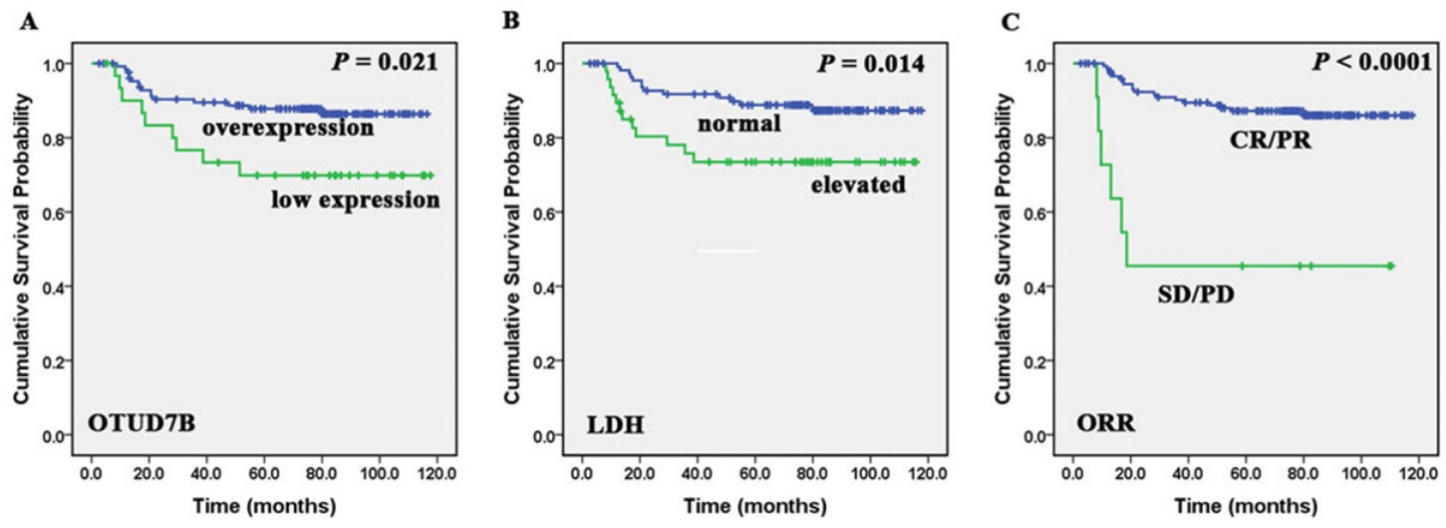
** Correlation of OTUD7B, LDH and ORR with DLBCL patients' overall survival.** Kaplan-Meier curves showing the association between OTUD7B expression (A), LDH levels (B), ORR (C) and OS in DLBCL patients. All the *P* values are shown in the graph, by log-rank test.

We found that low expression of OTUD7B was more detected in patients with more than one extranodal sites (*P*=0.006) and high IPI scores (*P*=0.022) (Table 1). Patients with overexpression of OTUD7B experienced better overall survival comparing to those with OTUD7B low expression (*P*=0.021). Besides, Kaplan-Meier analysis showed that patients with low LDH levels, achieved CR/PR after first-line treatment regimens had better OS (*P*=0.014, <0.0001) (Figure 2). A multivariate Cox proportional hazards model was performed to identify whether the protein showed prognostic significance compared with gender, age, ECOG, stage, LDH level, extranodal sites, IPI score, bulky disease, cell of origin and first-line treatment outcome. It indicated that overexpression of OTUD7B (*P* = 0.04, HR = 2.359, 95%CI: 1.039-5.358) was an independent prognostic factor (Table 2). We also analyzed PFS and OS in patients with different OTUD7B expression and their cell of origin, and found there was no statistically significant difference.

### OTUD7B expression levels and response to different chemotherapeutic agents

**Figure 3:**
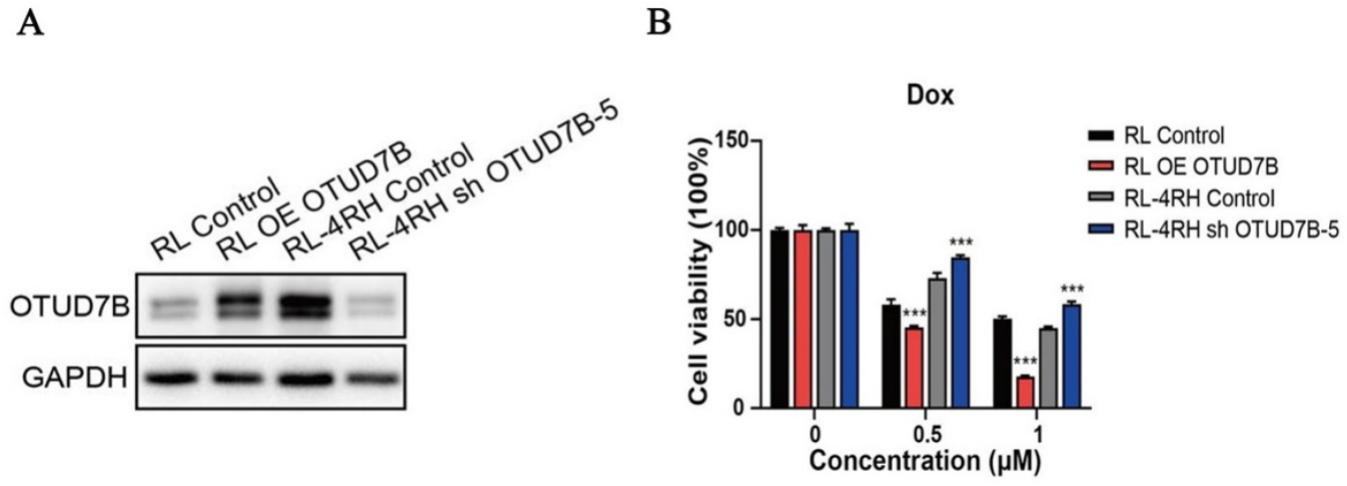
** OTUD7B is a positive regulator of dorubixin responsiveness in B-lymphoma cells.** (A) The overexpression and down-regulation of OTUD7B in RL and RL-4RH cells was examined by western blotting assay. (B) OTUD7B overexpressed RL cells and OTUD7B depleted RL-4RH cells were treated by 0.5μM or 1 μM, then the cell viability were monitored by MTT assays.

To study the role of OTUD7B in improving chemosensitivity, OTUD7B was stably overexpressed in a DLBCL cell line, resulting in the enhancement of cytotoxicity during doxorubicin treatment. Accordingly, Down-regulation of OTUD7B exhibited an opposite effect, which desensitized DLBCL cells to doxorubicin (Figure 3).

**Figure 4:**
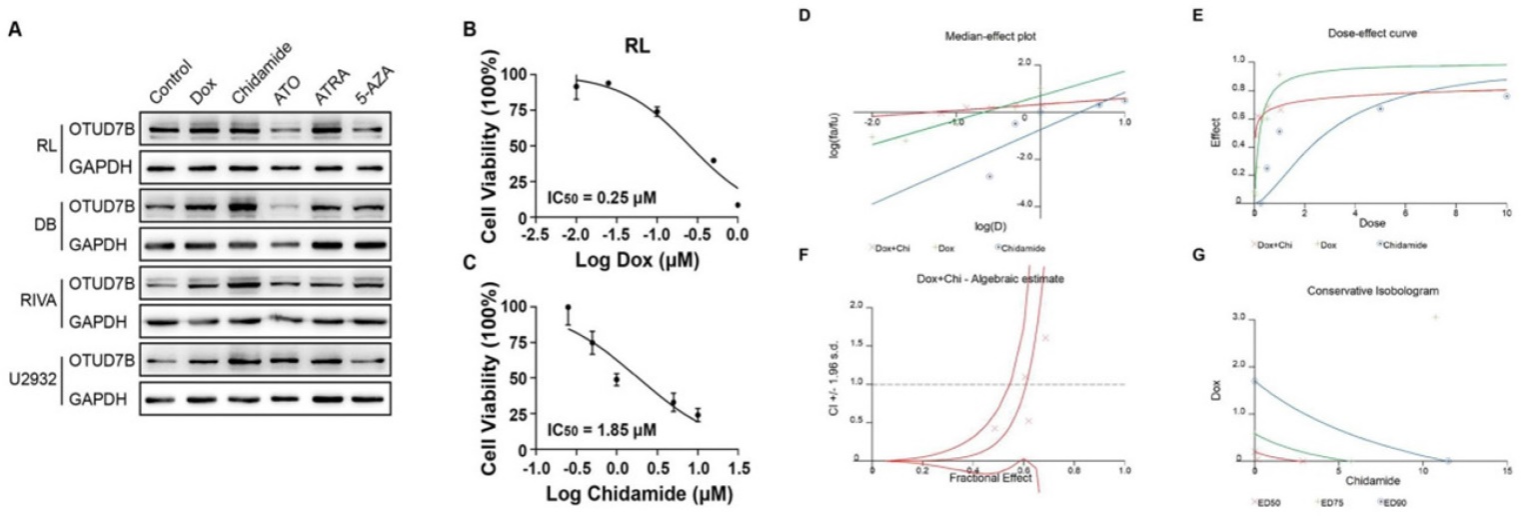
** An OTUD7B increasing reagent improves the efficiency of doxorubicin.** (A) Screening of chemotherapy reagents in effecting OTUD7B expression in multiple DLBCL cells. (B-C) IC50 of doxorubicin and chidamide in RL cell line. (D-G) Chidamide and doxorubicin had synergistic effect at low concentration.

In view of OTUD7B as a negative regulator of doxorubicin resistance, we hypothesized that there is a chemotherapeutic agent, which upregulates OTUD7B, may contribute to moderating doxorubicin irresponsiveness in DLBCL patients. To find an OTUD7B positive regulator, we screened a set of frequently-used DLBCL drugs for their effects in OTUD7B expression. The results showed that Chidamide remarkably up-regulated OTUD7B expression levels in several B-cell lymphoma cell lines (Figure 4A). Furthermore, we demonstrated that the combination of chidamide and doxorubicin revealed a synergistic effect in inhibiting growth at relatively low concentrations in B-cell lymphoma cell line (Figure 4B-G).

## Discussion

DLBCL is a heterogeneous disease in both clinical and biological settings. Identification of prognostic biomarkers is of vital importance. Increasingly evidences highlight the importance of OTUD7B in several types of human malignancies 10, 11. However, the clinical relevance of this protein in DLBCL is not entirely identified. Herein, we found that OTUD7B could serve as a novel biomarker indicative of favorable prognosis in DLBCL patients.

Although the molecular events underlying clinical significance of OTUD7B remain unclear, it is speculated by existing works. A recent study illustrated a role of OTUD7B in cell-cycle regulation, which could prevent the degradation of APC/C substrate, and lead to deregulation of mitotic process 12. More importantly, OTUD7B has also been reported to participate in NF-κB signaling pathways. It has long been known that NF-κB signaling is critical for DLBCL development and contributes to drug resistance. Hyperactivation of NF-κB is associated with interior prognosis 13-15. The activation of NF-κB requires the degradation of an inhibitory protein TRAF3. OTUD7B deubiquitinates and stabilizes TRAF3, attenuating the noncanonical NF-κB pathway 16. Therefore, OTUD7B down-regulation may accelerate DLBCL progression and counteracts chemotherapy through promoting NF-κB activity.

Further analysis showed that low expression of OTUD7B was associated with DLBCL patients having more than one extranodal sites and high IPI scores (*P*=0.006, 0.022), which was in consistent with other researches about this protein in solid malignant tumors. Downregulation of OTUD7B was reported to be associated with large tumor size, more satellite nodules and more vascular invasion in hepatocellular carcinoma and lung cancer 7, 17. Also there were studies demonstrated that interference of OTUD7B expression promoted the migration and invasion of cancer cells in vitro 18, 19. It could be easily explained that more sites of extranodal lesions, and high IPI score associated with worse OS of DLBCL patients 20, 21. Moreover, we carried on multivariate Cox regression analysis, and found that OTUD7B was an independent prognostic indicator in DLBCL patients.

Remarkably, the findings of this work provide a new insight in the treatment of DLBCL, that is, up-regulation of OTUD7B may synergize the anti-tumoral activity of doxorubicin. Functional studies showed that forced expression of OTUD7B sensitized B-cell lymphoma cell lines to doxorubicin. To extend this phenotype to a clinical actionable way, we screened a handful of drugs in increasing OTUD7B expression. The results showed that Chidamide is capable of elevating OTUD7B in several DLBCL cell lines. Chidamide is a histone deacetylase inhibitor of the benzamide class with profound antitumor activity in peripheral T cell lymphoma. It also has shown promising result in the treatment of DLBCL 22-24. *In vivo* pharmacological assays revealed that Chidamide cooperatively improved the inhibitory efficacy of doxorubicin. Taken together, our data shed light on a new treatment modality of DLBCL and OTUD7B might be a potential biomarker in deciding the use of chidamide.

In conclusion, our present data suggested that OTUD7B might be a novel anti-oncogene that has a pivotal role in DLBCL and contribute to the selection of patients who may benefit from chidamide-contained treatment therapy. Further investigations are still needed.

## Figures and Tables

**Table 1:** OTUD7B expression and basic clinical/laboratory features in 160 DLBCL patients

		OTUD7B Expression		*P*-value
	n	negative (%)	positive (%)	
Gender				
Female	71	17(23.9)	54(76.1)	0.192
Male	89	14(15.7)	75 (84.3)	
Age, years				
Median	51			
≤60	118	25(21.2)	93(78.8)	0.331
>60	42	6(14.3)	36(85.7)	
ECOG score				
0	100	20(20.0)	80(80.0)	0.796
1	60	11(18.3)	49(81.7)	
Ann Arbor Stage				
I	45	8(17.8)	37(82.2)	0.078
II	53	11(20.8)	42(79.2)	
III	36	3(8.3)	33(91.7)	
IV	26	9(34.6)	17(65.4)	
Number of extranodal sites				
0	83	16(19.3)	67(80.7)	0.006
1	56	6(10.7)	50(89.3)	
>1	21	9(42.9)	12(57.1)	
LDH level				
Normal (≤250)	113	20(17.7)	93(82.3)	0.406
>Normal	47	11(23.4)	36(76.6)	
IPI score				
0-1	108	19(17.6)	89(82.4)	0.022
2	33	4(12.1)	29(87.9)	
≥3	19	8(42.1)	11(57.9)	
B symptom				
yes	29	4(13.8)	25(86.2)	0.401
no	131	27(20.6)	104(79.4)	
Bulky disease				
yes	48	9(18.8)	39(81.3)	0.896
no	112	22(19.6)	90(80.4)	
Cell of Origen (n=142)				
GCB	64	9(14.1)	55(85.9)	0.315
non-GCB	78	16(20.5)	62(79.5)	

**Table 2:** Univariate and multivariate analysis of overall survival in DLBCL patients

Variable	Univariate Analysis			Multivariate Analysis		
	HR	95% CI	*P*	HR	95%CI (95%CI)	*P*
OTUD7B						
High vs. low expression	2.537	1.121-5.741	0.026	2.359	1.039-5.358	0.04
Age						
>60 vs. ≤60	1.869	0.839-4.163	0.126			
Gender						
Male vs. female	0.6	0.272-1.322	0.205			
ECOG						
0 vs. 1	1.568	0.715-3.438	0.261			
Stage						
I-II vs.III-IV	1.457	0.499-4.251	0.491			
LDH						
normal vs. elevated	2.583	1.177-5.665	0.018	2.069	0.921-4.649	0.078
Extranodal sites						
0-1 vs. ≥2	0.624	0.276-1.412	0.258			
IPI score						
0-1 vs. ≥2	1.820	0.825-4.011	0.138			
Bulky disease						
yes vs. no	1.071	0.462-2.481	0.873			
B symptoms						
yes vs. no	1.784	0.745-4.273	0.194			
Cell of origin						
GCB vs. non-GCB	1.709	0.697-4.191	0.242			
ORR						
CR/PR vs. SD/PD	6.595	2.625-16.572	<0.0001	4.962	1.917-12.848	0.001

HR = Hazard Ratio.

## References

[1] Kubuschok B, Held G, Pfreundschuh M (2015). Management of diffuse large B-cell lymphoma (DLBCL). Cancer treatment and research.

[2] Wang B, Jie Z, Joo D, Ordureau A, Liu P, Gan W (2017). TRAF2 and OTUD7B govern a ubiquitin-dependent switch that regulates mTORC2 signalling. Nature.

[3] Enesa K, Zakkar M, Chaudhury H, Luong le A, Rawlinson L, Mason JC (2008). NF-kappaB suppression by the deubiquitinating enzyme Cezanne: a novel negative feedback loop in pro-inflammatory signaling. J Biol Chem.

[4] Hu H, Wang H, Xiao Y, Jin J, Chang JH, Zou Q (2016). Otud7b facilitates T cell activation and inflammatory responses by regulating Zap70 ubiquitination. J Exp Med.

[5] Pareja F, Ferraro DA, Rubin C, Cohen-Dvashi H, Zhang F, Aulmann S (2012). Deubiquitination of EGFR by Cezanne-1 contributes to cancer progression. Oncogene.

[6] Chiu HW, Lin HY, Tseng IJ, Lin YF (2018). OTUD7B upregulation predicts a poor response to paclitaxel in patients with triple-negative breast cancer. Oncotarget.

[7] Wang JH, Wei W, Guo ZX, Shi M, Guo RP (2015). Decreased Cezanne expression is associated with the progression and poor prognosis in hepatocellular carcinoma. J Transl Med.

[8] Zhang B, Wang H, Yang L, Zhang Y, Wang P, Huang G (2016). OTUD7B and NIK expression in non-small cell lung cancer: Association with clinicopathological features and prognostic implications. Pathol Res Pract.

[9] Czuczman MS, Olejniczak S, Gowda A, Kotowski A, Binder A, Kaur H (2008). Acquirement of rituximab resistance in lymphoma cell lines is associated with both global CD20 gene and protein down-regulation regulated at the pretranscriptional and posttranscriptional levels. Clinical cancer research: an official journal of the American Association for Cancer Research.

[10] Nikolaou K, Tsagaratou A, Eftychi C, Kollias G, Mosialos G, Talianidis I (2012). Inactivation of the deubiquitinase CYLD in hepatocytes causes apoptosis, inflammation, fibrosis, and cancer. Cancer Cell.

[11] L'Esperance S, Popa I, Bachvarova M, Plante M, Patten N, Wu L (2006). Gene expression profiling of paired ovarian tumors obtained prior to and following adjuvant chemotherapy: molecular signatures of chemoresistant tumors. Int J Oncol.

[12] Bonacci T, Suzuki A, Grant GD, Stanley N, Cook JG, Brown NG (2018). Cezanne/OTUD7B is a cell cycle-regulated deubiquitinase that antagonizes the degradation of APC/C substrates. EMBO J.

[13] Skunca Z, Planinc-Peraica A (2015). [PROGNOSTIC ROLE OF NF-kappaB EXPRESSION IN DIFFUSE LARGE B-CELL LYMPHOMA SUBGROUPS]. Acta Med Croatica.

[14] Jost PJ, Ruland J (2007). Aberrant NF-kappaB signaling in lymphoma: mechanisms, consequences, and therapeutic implications. Blood.

[15] McNally RS, Davis BK, Clements CM, Accavitti-Loper MA, Mak TW, Ting JP (2011). DJ-1 enhances cell survival through the binding of Cezanne, a negative regulator of NF-kappaB. J Biol Chem.

[16] Hu H, Brittain GC, Chang JH, Puebla-Osorio N, Jin J, Zal A (2013). OTUD7B controls non-canonical NF-kappaB activation through deubiquitination of TRAF3. Nature.

[17] Pang Z, Cui L, Ding N, Zhu L, Qu X, Dong W (2017). Expressions of insulin-like growth factor receptor-1 and cezanne-1 in lung adenocarcinoma. Med Oncol.

[18] Wang JH, Zhong XP, Zhang YF, Wu XL, Li SH, Jian PE (2017). Cezanne predicts progression and adjuvant TACE response in hepatocellular carcinoma. Cell Death Dis.

[19] Lin DD, Shen Y, Qiao S, Liu WW, Zheng L, Wang YN (2019). Upregulation of OTUD7B (Cezanne) Promotes Tumor Progression via AKT/VEGF Pathway in Lung Squamous Carcinoma and Adenocarcinoma. Front Oncol.

[20] Ruppert AS, Dixon JG, Salles G, Wall A, Cunningham D, Poeschel V (2020). International prognostic indices in diffuse large B-cell lymphoma: a comparison of IPI, R-IPI, and NCCN-IPI. Blood.

[21] Yao S, Li J, Yao Z, Xu Y, Chu J, Zhang J (2017). Extranodal involvement in young patients with diffuse large B-cell lymphoma: distribution, prognostic value and treatment options. Chin J Cancer Res.

[22] Li Q, Huang J, Ou Y, Li Y, Wu Y (2019). Progressive diffuse large B-cell lymphoma with TP53 gene mutation treated with chidamide-based chemotherapy. Immunotherapy.

[23] Zhang MC, Fang Y, Wang L, Cheng S, Fu D, He Y (2020). Clinical efficacy and molecular biomarkers in a phase II study of tucidinostat plus R-CHOP in elderly patients with newly diagnosed diffuse large B-cell lymphoma. Clin Epigenetics.

[24] Guan XW, Wang HQ, Ban WW, Chang Z, Chen HZ, Jia L (2020). Novel HDAC inhibitor Chidamide synergizes with Rituximab to inhibit diffuse large B-cell lymphoma tumour growth by upregulating CD20. Cell Death Dis.

